# Supplementary material for: Modeling using clinical examination indicators predicts interstitial lung disease among patients with rheumatoid arthritis
Source: PeerJ. 2017 Feb 21;5:e3021. doi: 10.7717/peerj.3021 (PMC5322753; doi:10.7717/peerj.3021)
Supplement: Table S4 [file peerj-05-3021-s004.docx]

**Table S4**. The 2×2 contingency table of validation set.

| total population | ILD (predicted) | Healthy (predicted) | sum |
| --- | --- | --- | --- |
| ILD (true) | 124 (TP) | 22 (FN) | 146 (TP+FN) |
| Healthy (true) | 27 (FP) | 45 (TN) | 72 (FP+TN) |
| sum | 151 (TP+FP) | 67 (FN+TN) | 218 (TP+FP+FN+TN) |

TP: true positive; FN: false negative; FP: false positive; TN: true negative.
